# ^1^H, ^13^C and ^15^N backbone resonance assignment of the lytic polysaccharide monooxygenase *Ls*AA9A from *Lentinus similis*

**DOI:** 10.1007/s12104-025-10256-z

**Published:** 2025-11-25

**Authors:** Piera Wiesinger, Mats Sandgren, Gustav Nestor

**Affiliations:** Department of Molecular Sciences, Swedish University of Agricultural Sciences, Uppsala, Sweden

**Keywords:** Cellooligomers, Cellulose, Lytic polysaccharide monooxygenase (LPMO), NMR, *Ls*AA9A

## Abstract

Lytic polysaccharide monooxygenases (LPMOs) are mono-copper binding enzymes involved in the degradation of carbohydrates. The 25 kDa sized LPMO *Ls*AA9A from the basidiomycete *Lentinus similis* is known to oxidate cellulose and cellooligomers at the C4 position and thus leading to a breakage of the glycosidic bond. *Ls*AA9A has been recombinantly expressed in *Escherichia coli* with ^13^C and ^15^N labelling. Here, we present the ^1^H, ^13^C and ^15^N backbone resonance assignment of the *apo* form. The secondary structure was predicted using the TALOS-N software and it was overall in agreement with the crystal structure of *Ls*AA9A expressed in *E. coli*. A few shorter α-helices and β-sheets present in the crystal structure are missing in the NMR prediction and vice versa. *Ls*AA9A resembles the typical structural elements of LPMOs with a core β-sandwich.

## Biological context

Lytic polysaccharide monooxygenases (LPMOs) are carbohydrate-degrading enzymes predominantly found in fungi and bacteria, but also in viruses, plants and insects (Vandhana et al. 2022). LPMOs cleave glycosidic bonds in an oxidative manner via a mono-copper coordinated by a histidine brace in the active site (Bissaro and Eijsink 2023). The common structure of LPMOs is a core β-sheet sandwich and a few short α-helices connected via loops. LPMOs have a characteristic flat substrate-interaction surface, which also includes the catalytic site. This feature enables LPMOs to act on crystalline polysaccharides such as cellulose or chitin (Aachmann et al. 2012), but they can also be active on shorter poly- and oligosaccharides (Frandsen et al. 2016; Rieder et al. 2021). The described features are crucial for the ability of LPMOs to degrade carbohydrates both for industrial applications as well as in their biological context. Since the first crystal structures of LPMOs (Karkehabadi et al. 2008; Vaaje-Kolstad et al. 2005b) and their boosting effect on the degradation efficiency of cellulose (Harris et al. 2010) and chitin (Vaaje-Kolstad et al. 2005a, ) was revealed about 20 years ago, much research has been focused on industrial applications. More recently, it has been shown that LPMOs play biological roles in pathogenesis, cell wall remodelling and copper transport (Vandhana et al. 2022).

The LPMO *Ls*AA9A from the basidiomycete *Lentinus similis* has been characterized in depth both biochemically and structurally (Frandsen et al. 2016; Simmons et al. 2017; Tandrup et al. 2023). It is capable of oxidizing cellulose and cellooligomers at the C4 position and other polysaccharides at both the C1 and C4 position (Simmons et al. 2017). The crystal structure of *Ls*AA9A was first solved by Frandsen et al. (2016), who also presented complex structures with cellooligomers bound. These structures have been used as the basis for mechanistic studies such as investigations on the copper coordination sphere upon reduction (Tandrup et al. 2022) or computational studies on the electronic effect of priming reduction, substrate and co-substrate binding (Wieduwilt et al. 2024).

Five catalytic domains of LPMOs, including three bacterial (Aachmann et al. 2011; Christensen et al. 2021; Courtade et al. 2015) and two fungal (Courtade et al. 2016; Kitaoku et al. 2018) LPMOs, have been expressed with isotopic labelling for NMR studies and their backbone resonance assignments have been deposited in BMRB to date. However, no data has been deposited for *Ls*AA9A so far.

In this study, we present the recombinant expression and purification of ^13^C and ^15^N labelled *Ls*AA9A and a nearly complete backbone resonance assignment of *apo*-*Ls*AA9A. Four residues which are located in a loop in the crystal structure could not be assigned, probably due to intermediate dynamics, making them invisible on the NMR time scale. Additionally, we present a secondary structure prediction using the TALOS-N software. The secondary elements predicted from the NMR data are to a large extent coherent with the crystallographic data. The crystal structure (PDB code 7PQR) displays two *α*-helices which are absent in the NMR prediction, whereas the predicted NMR structure contains a few additional short *β*-strands.

## Methods and experiments

### Protein expression and purification

*Ls*AA9A was recombinantly expressed by a BL21(DE3) *E. coli* strain containing the plasmid pLyGo-Ec-6_*Ls*AA9A (Hernández-Rollán et al. 2021; Tandrup et al. 2022). The expression was done in M9 minimal media for isotopic labelling purposes using 1 g/l NH_4_Cl (99% ^15^N from Cortecnet, Les Ulis, France) and 2 g/l D-glucose (99% U-^13^C from Cambrige Isotope Laboratories, Inc, Andover, MA, USA). A 5 ml pre-culture in LB medium was incubated for 6 h at 37 °C shaking at 250 rpm. This pre-culture was transferred to 50 ml M9 medium incubating at the same conditions for ~ 14 h. For the protein expression 1 l of M9 minimal medium was inoculated with the 50 ml culture and grown until the OD_600_ was ~ 0.8 at 37 °C with shaking at 180 rpm. The induction was done with 1 mM (final concentration) isopropyl β-d-1-thiogalactopyranoside (IPTG) and the protein was expressed for ~ 20 h at 16 °C shaking at 150 rpm. The cells were harvested by centrifugation (4000 rpm, 20 min). The cell pellet was re-suspended in 35 ml 25 mM Bis–Tris buffer pH 5.9 and stored at −20 °C until further usage.

For protein extraction the suspended cells were incubated with DNase (10 µg/ml) for 1 h gently shaking on ice before and after cell disruption. The cells were disrupted with a Cell Disruptor (Constant Systems Ltd, Daventry, United Kingdom) at 20 kPsi and centrifuged at 18,000 rpm for 40 min. The supernatant was filtered with a 0.45 µm filter.

For purification of *Ls*AA9A, an ÄKTApurifier system and columns from Cytiva (Uppsala, Sweden) were used. The protein extract was desalted with a HiPrep 26/10 Desalting column using a 25 mM Bis–Tris buffer pH 5.9. This step was followed by anion-exchange chromatography purification with 2 × 5 ml HiTrap Q HP columns. The running buffers used were: (A) 25 mM Bis–Tris, pH 5.9 and (B) 25 mM Bis–Tris, 1 M NaCl, pH 5.9. The following gradient was used with a flow rate of 3 ml/min: 0–70 ml sample loading with buffer A, 150 ml linear gradient to 50% buffer B, 50 ml linear gradient to 100% buffer B, 50 ml buffer B, 60 ml buffer A for column regeneration. Fractions containing *Ls*AA9A were pooled, concentrated with a Vivaspin 5 MWCO centrifugal concentrator and filtered with a 0.25 µm filter before loading on a size exclusion column (SEC) (Superdex 75 16/600). The SEC running buffer used was 40 mM NaOAc, 100 mM NaCl, pH 5.9. All buffers were filtered and degassed with 0.45 µm filters before use. Fractions from the SEC purification were analysed by SDS-PAGE for purity determination. Protein concentrations were determined spectrophotometrically at 280 nm with an extinction coefficient of 48,025 M^−1^ cm^−1^.

For the NMR sample the purest *Ls*AA9A fractions were combined and concentrated to 300 µM. The buffer was exchanged to 40 mM sodium phosphate, 10 mM NaCl, pH 6. Before transferring the sample to a Shigemi NMR tube 0.02% NaN_3_ and 10% D_2_O were added, as well as 0.5 mM DSS for chemical shift referencing.

### NMR spectroscopy

All NMR experiments were performed at the Swedish NMR centre in Gothenburg on a 900 MHz spectrometer (Oxford 900 magnet with Bruker Avance HDIII console) equipped with a 3 mm TCI ^1^H/^13^C/^15^N CryoProbe. For ^1^H, ^13^C and ^15^N backbone resonance assignment data was collected with the following experiments: 1D 1H, 2D ^1^H,^15^N-HSQC and 3D HNCO, HN(CA)CO, HNCA, HN(CO)CA, HNCACB, CBCA(CO)NH and HBHA(CO)NH. All experiments are standard experiments in the Bruker library. The spectra were recorded at 37 °C with 20–25% non-uniform sampling (NUS) and reconstructed and processed with TopSpin 4.1.4. ^1^H chemical shifts were referenced to DSS (δ = 0.00 ppm) and ^13^C and ^15^N chemical shifts were indirectly referenced according to Wishart et al. (1995). The assignment was performed manually with CcpNmr AnalysisAssign Version 3 (Skinner et al. 2016) and with the input of NMRtist (Klukowski et al. 2022, 2023) chemical shift results (NMRtist calculations performed on 2024–02–22). Predictions from the SHIFTX2 software (Han et al. 2011) were used for assistance.

### Secondary structure prediction

Secondary structure elements and the random coil index order parameter S^2^ (RCI) (Berjanskii & Wishart 2005, 2008) were predicted with the TALOS-N program (Shen & Bax 2013). The web-based version of the software was used (https://spin.niddk.nih.gov/bax-apps/nmrserver/talosn/). The predictions are based on backbone resonance chemical shift assignment data including also Hα. It was compared to the crystal structure of *Ls*AA9A expressed in *E. coli* (PDB: 7PQR) (Tandrup et al. 2022).

### Extent of assignment and data deposition

#### Backbone resonance assignment of *Ls*AA9A

**Fig. 1:**
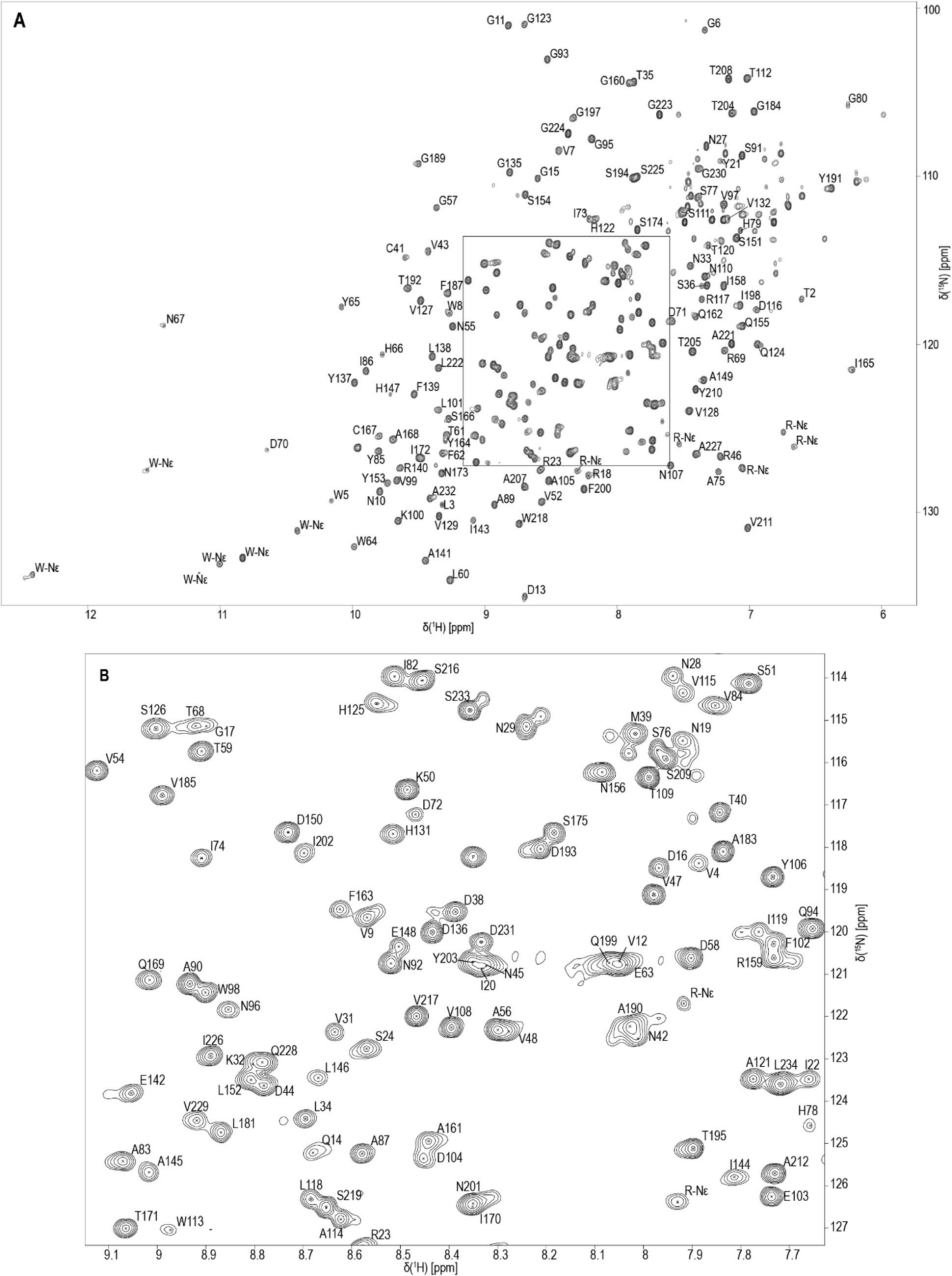
^1^H,^15^N-HSQC spectrum of *Ls*AA9A (**A**) with an expansion of the middle region (**B**) shown below. The spectrum was acquired from a sample of 300 µM ^13^C,^15^N-*Ls*AA9A in 40 mM sodium phosphate, 10 mM NaCl, pH 6 and 10% D_2_O at 37 °C on a 900 MHz spectrometer

We present the assignment of backbone resonances of the copper-free form (*apo*-form) of AA9A from *Lentinus similis*. In earlier experiments, the binding of copper by addition of copper acetate and removing of copper by EDTA was tested. These experiments confirmed that *Ls*AA9A is predominantly without copper after expression in *E. coli* and purification. The well dispersed and sharp cross-peaks in the 2D ^1^H,^15^N-HSQC indicate a folded enzyme (Fig. 1) which is stable even after several days of data collection at 37 °C. The protein sequence contains 235 amino acid residues of which 20 are prolines. The backbone assignment is to > 84% complete (^1^HN 97%, ^15^N 89%, ^13^Cα 88%, ^13^Cβ 86%, ^13^CO 84% and ^1^Hα 87%). For most prolines the C´, Cα, Cβ and Hα could be assigned. The backbone resonances of six other residues could not be assigned. This includes A133, G220 and residues D176, D177, S178 and T179. G220 is predicted by the SHIFTX2 software to have its HN proton chemical shift near the water signal (~ 5 ppm), which is most likely the reason why it could not be detected. Residue 133 and 176 to 179 are located in loops in the crystal structure (7PQR) in close proximity to each other. We therefore suspect that they are flexible in a dynamic range where a signal could not be detected by NMR spectroscopy. The cross-peaks which we expect to correspond to side-chain NH resonances of six tryptophan and seven arginine residues have been marked as W/R-Nε respectively (Fig. 1). The chemical shift data including N, HN, C´, Cα, Cβ, Hα and Hβ has been deposited at the Biological Magnetic Resonance Bank (BMRB), with the accession code 53342.

#### Secondary structure of *Ls*AA9A in solution

**Fig. 2:**
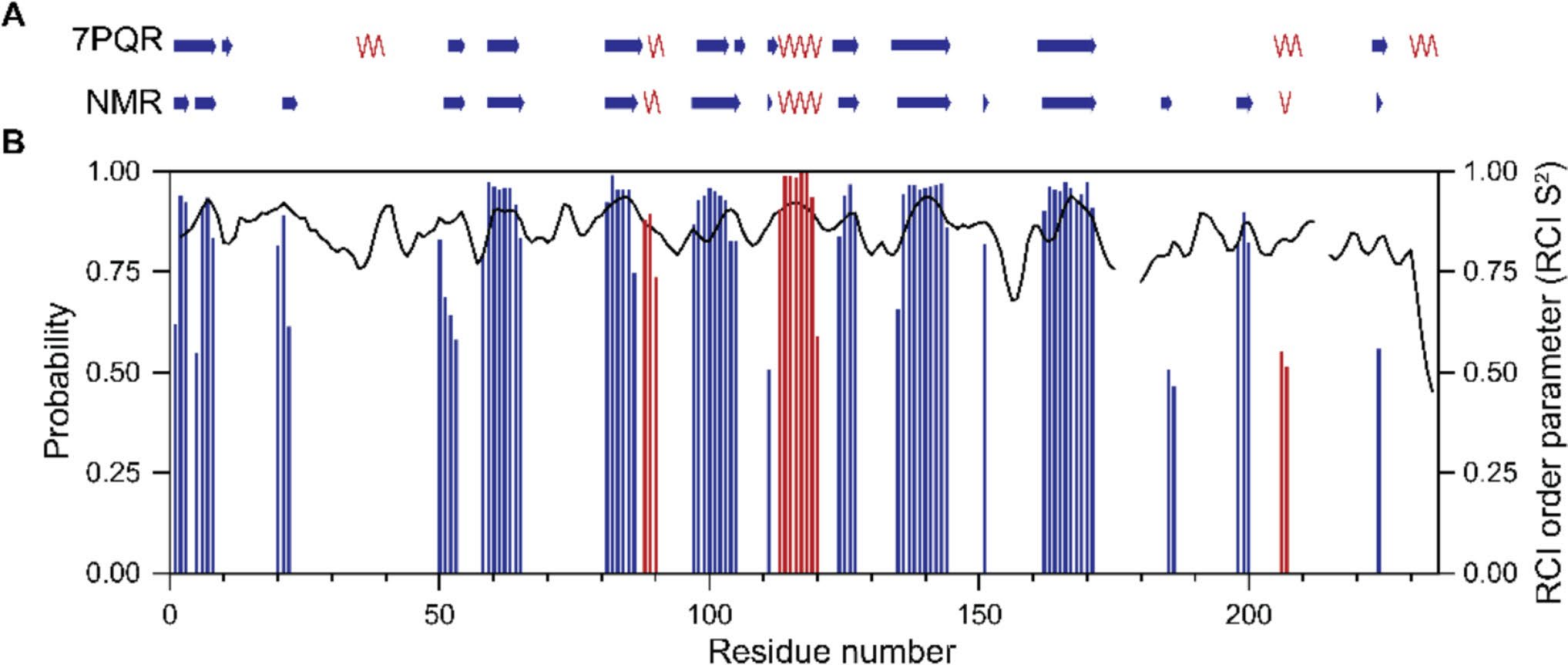
Secondary structure elements of *apo*-*Ls*AA9A predicted by TALOS-N: (**A**) schematic comparison of α-helices (red) and β-sheets (blue) observed in the crystal structure (7PQR) and predicted from NMR backbone resonance assignment data, (**B**) probability of structural elements (α-helices (red bars) and β-sheets (blue bars)) and random coil index (RCI) order parameter S^2^ (black line) for each residue

The protein backbone torsion angles and secondary structure of *Ls*AA9A in solution were predicted by the TALOS-N software package. In Fig. 2 the structural elements in the crystal structure (PDB code: 7PQR) and NMR prediction, as well as the probability of a predicted structural element and the random coil index order parameter S^2^ are shown.

**Fig. 3:**
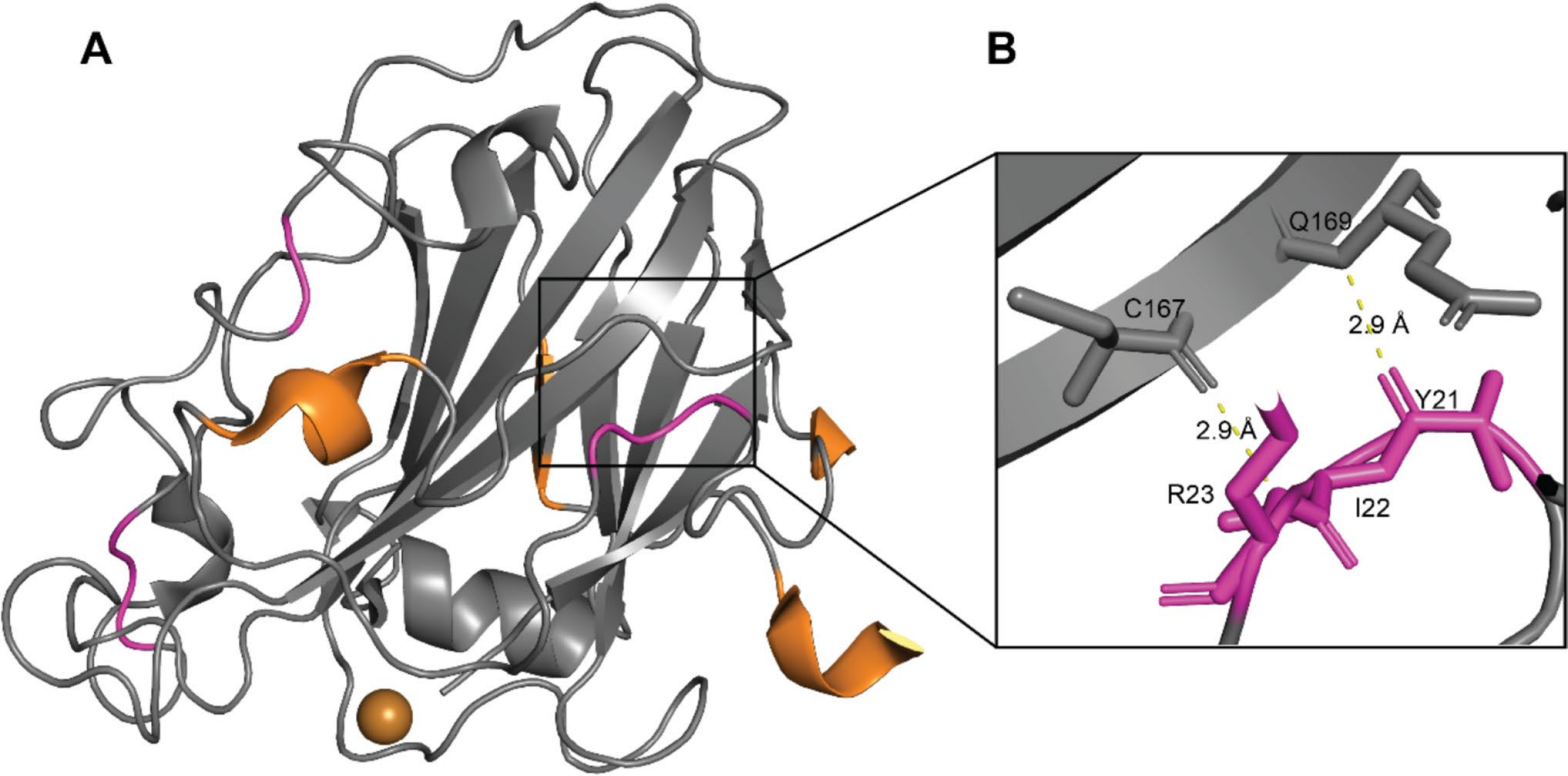
Crystal structure (7PQR) with highlighted differences compared to NMR secondary structure prediction. The copper ion (gold) is present in the *Ls*AA9A crystals, but not in the NMR sample. (**A**) α-helices and β-strands observed in the X-ray crystal structure, but not in the NMR structure prediction are marked in orange. Loops in the crystal structure predicted as β-strands from the NMR data are marked in magenta. (**B**) Zoom-in on potential β-strand formation of residues 21–23 and interaction with the major β-sheet, specifically hydrogen bonding (N(H)-CO 2.9 Å) to residues 167–169

Overall, the structural elements of *Ls*AA9A are coherent between NMR data and the X-ray crystal structure. Some minor differences are observed in the length or exact start of α-helices or β-sheets. However, also some major differences were observed and visualized in Fig. 3. Additional to the NMR prediction, the X-ray structure contains a short β-strand at position 12 and 13 as being antiparallel to the previous β-sheets. In contrast, the NMR prediction contains a β-strand at position 21 to 23, which is absent in the X-ray structure. The N(H)-CO distance to the neighbouring major β-sheet in the crystal structure is 2.9 Å. This distance is also observed between other β-sheets in the enzyme. We therefore conclude that the residues 21 to 23 can form a short β-strand interacting with residues 167–169. The crystal structure shows a helix from position 36 to 40, which is not predicted from the NMR data. At position 186/187 and 199–201 TALOS-N predicts two β-strands, which are absent in the crystal structure. Both the following α-helix in the crystal structure at position 206–210 and β-strand at 224–226 are much shorter in the NMR secondary structure prediction. The very last α-helix from position 231 to 235 is absent in the NMR structure and marked as dynamic by TALOS-N. Some of the predictions, such as residues 186/187 and 224–226, have a probability of just above the TALOS-N threshold of 0.5. Therefore, the prediction should be taken with caution.

All differences of the secondary elements between NMR data and X-ray crystal structure are located outside the core β-sandwich and outside the substrate binding site. The distance to the copper binding site is at least 15 Å. It should be pointed out that the NMR data was collected on the *apo*-form (copper free) protein and X-ray data on the *holo*-form (copper bound). However, due to the lack of proximity to the copper we believe that the differences in secondary structure cannot be related to the absence or presence of copper, but rather to the protein being in a dynamic or static environment.

## Data Availability

The backbone resonance assignment data was deposited at the BMRB under the accession number 53342.

## References

[CR1] Aachmann FL, Eijsink VG, Vaaje-Kolstad G (2011) 1H, 13C, 15N resonance assignment of the chitin-binding protein CBP21 from serratia marcescens. Biomol NMR Assign 5(1):117–119. 10.1007/s12104-010-9281-2 21052875 10.1007/s12104-010-9281-2

[CR2] Aachmann FL, Sørlie M, Skjåk-Bræk G, Eijsink VG, Vaaje-Kolstad G (2012) NMR structure of a lytic polysaccharide monooxygenase provides insight into copper binding, protein dynamics, and substrate interactions. Proc Natl Acad Sci U S A 109(46):18779–18784. 10.1073/pnas.1208822109 23112164 10.1073/pnas.1208822109 PMC3503203

[CR3] Berjanskii MV, Wishart DS (2005) A simple method to predict protein flexibility using secondary chemical shifts. J Am Chem Soc 127(43):14970–14971. 10.1021/ja054842f 16248604 10.1021/ja054842f

[CR4] Berjanskii MV, Wishart DS (2008) Application of the random coil index to studying protein flexibility. J Biomol NMR 40(1):31–48. 10.1007/s10858-007-9208-0 17985196 10.1007/s10858-007-9208-0

[CR5] Bissaro B, Eijsink VGH (2023) Lytic polysaccharide monooxygenases: enzymes for controlled and site-specific Fenton-like chemistry. Essays Biochem 67(3):575–584. 10.1042/ebc20220250 36734231 10.1042/EBC20220250 PMC10154617

[CR6] Christensen IA, Eijsink VG, Aachmann FL, Courtade G (2021) ^1^H, ^13^C, ^15^N resonance assignment of the apo form of the small, chitin-active lytic polysaccharide monooxygenase JdLPMO10A from *Jonesia denitrificans*. Biomol NMR Assign 15(1):79–84. 10.1007/s12104-020-09986-z 33215349 10.1007/s12104-020-09986-z

[CR7] Courtade G, Balzer S, Forsberg Z, Vaaje-Kolstad G, Eijsink VGH, Aachmann FL (2015) 1H, 13C, 15N resonance assignment of the chitin-active lytic polysaccharide monooxygenase BlLPMO10A from *Bacillus licheniformis*. Biomol NMR Assign 9(1):207–210. 10.1007/s12104-014-9575-x 25204609 10.1007/s12104-014-9575-x

[CR8] Courtade G, Wimmer R, Dimarogona M, Sandgren M, Eijsink VG, Aachmann FL (2016) Backbone and side-chain 1H, 13C, and 15N chemical shift assignments for the apo-form of the lytic polysaccharide monooxygenase NcLPMO9C. Biomol NMR Assign 10(2):277–280. 10.1007/s12104-016-9683-x 27147444 10.1007/s12104-016-9683-x

[CR9] Frandsen KE, Simmons TJ, Dupree P, Poulsen JC, Hemsworth GR, Ciano L, Johnston EM, Tovborg M, Johansen KS, von Freiesleben P, Marmuse L, Fort S, Cottaz S, Driguez H, Henrissat B, Lenfant N, Tuna F, Baldansuren A, Davies G, Walton JPH (2016) The molecular basis of polysaccharide cleavage by lytic polysaccharide monooxygenases. Nat Chem Biol 12(4):298–303. 10.1038/nchembio.2029 26928935 10.1038/nchembio.2029 PMC4817220

[CR10] Han B, Liu Y, Ginzinger SW, Wishart DS (2011) SHIFTX2: significantly improved protein chemical shift prediction. J Biomol NMR 50(1):43–57. 10.1007/s10858-011-9478-4 21448735 10.1007/s10858-011-9478-4 PMC3085061

[CR11] Harris PV, Welner D, McFarland KC, Re E, Navarro Poulsen J-C, Brown K, Salbo R, Ding H, Vlasenko E, Merino S, Xu F, Cherry J, Larsen S, Lo Leggio L (2010) Stimulation of lignocellulosic biomass hydrolysis by proteins of glycoside hydrolase family 61: structure and function of a large, enigmatic family. Biochemistry 49(15):3305–3316. 10.1021/bi100009p 20230050 10.1021/bi100009p

[CR12] Hernández-Rollán C, Falkenberg KB, Rennig M, Bertelsen AB, Ipsen JØ, Brander S, Daley DO, Johansen KS, Nørholm MHH (2021) LyGo: a platform for rapid screening of lytic polysaccharide monooxygenase production. ACS Synth Biol 10(4):897–906. 10.1021/acssynbio.1c00034 33797234 10.1021/acssynbio.1c00034

[CR13] Karkehabadi S, Hansson H, Kim S, Piens K, Mitchinson C, Sandgren M (2008) The first structure of a glycoside hydrolase family 61 member, cel61b from *Hypocrea jecorina*, at 1.6 å resolution. J Mol Biol 383(1):144–154. 10.1016/j.jmb.2008.08.016 18723026 10.1016/j.jmb.2008.08.016

[CR14] Kitaoku Y, Courtade G, Petrović DM, Fukamizo T, Eijsink VG, Aachmann FL (2018) Resonance assignments for the apo-form of the cellulose-active lytic polysaccharide monooxygenase ta LPMO9A. Biomol NMR Assign 12:357–361. 10.1007/s12104-018-9839-y 30117034 10.1007/s12104-018-9839-y

[CR15] Klukowski P, Riek R, Güntert P (2022) Rapid protein assignments and structures from raw NMR spectra with the deep learning technique ARTINA. Nat Commun 13(1):6151. 10.1038/s41467-022-33879-5 36257955 10.1038/s41467-022-33879-5 PMC9579175

[CR16] Klukowski P, Riek R, Güntert P (2023) NMRtist: an online platform for automated biomolecular NMR spectra analysis. Bioinformatics. 10.1093/bioinformatics/btad066 10.1093/bioinformatics/btad066 PMC9913044 36723167

[CR17] Rieder L, Petrović D, Valjamae P, Eijsink VG, Sørlie M (2021) Kinetic characterization of a putatively chitin-active LPMO reveals a preference for soluble substrates and absence of monooxygenase activity. ACS Catal 11(18):11685–11695. 10.1021/acscatal.1c03344 34567832 10.1021/acscatal.1c03344 PMC8453653

[CR18] Shen Y, Bax A (2013) Protein backbone and sidechain torsion angles predicted from NMR chemical shifts using artificial neural networks. J Biomol NMR 56(3):227–241. 10.1007/s10858-013-9741-y 23728592 10.1007/s10858-013-9741-y PMC3701756

[CR19] Simmons TJ, Frandsen KEH, Ciano L, Tryfona T, Lenfant N, Poulsen JC, Wilson LFL, Tandrup T, Tovborg M, Schnorr K, Johansen KS, Henrissat B, Walton PH, Lo Leggio L, Dupree P (2017) Structural and electronic determinants of lytic polysaccharide monooxygenase reactivity on polysaccharide substrates. Nat Commun 8(1):1064. 10.1038/s41467-017-01247-3 29057953 10.1038/s41467-017-01247-3 PMC5651836

[CR20] Skinner SP, Fogh RH, Boucher W, Ragan TJ, Mureddu LG, Vuister GW (2016) Ccpnmr analysisassign: a flexible platform for integrated NMR analysis. J Biomol NMR 66(2):111–124. 10.1007/s10858-016-0060-y 27663422 10.1007/s10858-016-0060-y PMC5095159

[CR21] Tandrup T, Muderspach SJ, Banerjee S, Santoni G, Ipsen JO, Hernandez-Rollan C, Norholm MHH, Johansen KS, Meilleur F, Lo Leggio L (2022) Changes in active-site geometry on X-ray photoreduction of a lytic polysaccharide monooxygenase active-site copper and saccharide binding. IUCrJ 9(Pt 5):666–681. 10.1107/S2052252522007175 10.1107/S2052252522007175 PMC9438499 36071795

[CR22] Tandrup T, Lo Leggio L, Meilleur F (2023) Joint X-ray/neutron structure of *Lentinus similis* AA9_a at room temperature. Acta Crystallogr Sect F Struct Biol Commun. 10.1107/S2053230X22011335 10.1107/S2053230X22011335 PMC9813973 36598350

[CR23] Vaaje-Kolstad G, Horn SJ, van Aalten DMF, Synstad B, Eijsink VGH (2005a) The non-catalytic chitin-binding protein CBP21 from serratia marcescensis essential for chitin degradation. J Biol Chem 280(31):28492–28497. 10.1074/jbc.M504468200 15929981 10.1074/jbc.M504468200

[CR24] Vaaje-Kolstad G, Houston DR, Riemen AHK, Eijsink VGH, van Aalten DMF (2005b) Crystal structure and binding properties of the *serratia marcescens* chitin-binding protein CBP21. J Biol Chem 280(12):11313–11319. 10.1074/jbc.M407175200 15590674 10.1074/jbc.M407175200

[CR25] Vandhana TM, Reyre JL, Sushmaa D, Berrin JG, Bissaro B, Madhuprakash J (2022) On the expansion of biological functions of lytic polysaccharide monooxygenases. New Phytol 233(6):2380–2396. 10.1111/nph.17921 34918344 10.1111/nph.17921

[CR26] Wieduwilt EK, Lo Leggio L, Hedegård ED (2024) A frontier-orbital view of the initial steps of lytic polysaccharide monooxygenase reactions. Dalton Trans 53(13):5796–5807. 10.1039/D3DT04275H 38445349 10.1039/d3dt04275h

[CR27] Wishart DS, Bigam CG, Yao J, Abildgaard F, Dyson HJ, Oldfield E, Markley JL, Sykes BD (1995) 1H, 13C and 15N chemical shift referencing in biomolecular NMR. J Biomol NMR 6(2):135–140. 10.1007/BF00211777 8589602 10.1007/BF00211777

